# Willingness to pay for residential property-based and community-based tick control methods in Lyme disease-endemic areas of the Upper Midwest, United States

**DOI:** 10.1016/j.ttbdis.2025.102531

**Published:** 2025-08-06

**Authors:** Elizabeth K. Schiffman, Jenna Bjork, Daniel Phaneuf, Alyssa Beck, Erik Foster, Jean I. Tsao, Rebecca Osborn, Rebecca Eisen, Susan Paskewitz, Sarah A. Hook, Alison F. Hinckley

**Affiliations:** a Minnesota Department of Health, 625 Robert Street North, Saint Paul, MN 55155, USA; b University of Wisconsin-Madison, 1656 Linden Drive, Madison, WI 53706; c Centers for Disease Control and Prevention, Division of Vector-Borne Diseases, 3156 Rampart Road, Fort Collins, CO 80521, USA; d Department of Fisheries and Wildlife and Department of Large Animal Clinical Sciences, Michigan State University, 480 Wilson Road, Natural Resources Building, East Lansing, MI 48824, USA; e Wisconsin Department of Health Services, 1 West Wilson Street, Madison, WI 53703, USA

**Keywords:** Lyme disease, Ticks, Tickborne diseases, Control, Willingness to pay, Upper Midwest

## Abstract

Ticks and tickborne diseases are of increasing concern in the United States, and the burden is high in certain focal areas. While the acceptability of various tick control and disease prevention methods has been studied, the public’s willingness to pay for environmental interventions at the individual or community level is less well described. Using data collected as part of a larger survey, we performed an additional analysis of residents of Lyme disease-endemic counties of Michigan, Minnesota, and Wisconsin to assess their willingness to support and pay annually for various methods of property-based tick control and examined demographic characteristics that might influence willingness to pay. Seventy-nine percent of respondents were willing to perform some form of tick control on their property, with most preferring self-application. Landscaping and natural pesticide application were the most popular options, with people willing to pay an estimated $78 and $61 annually, respectively. High income, a high perceived prevalence of disease, and a high perceived likelihood of disease were all associated with a willingness to pay more. When asked about a community control option, 97 % of respondents indicated interest, with respondents being willing to pay $52/year for a community-based program regardless of household characteristics. These results suggest a moderate demand in the Upper Midwest for tick control efforts at both the individual property level and for local, publicly funded, community-based programs. These findings provide a starting point for assessing community characteristics, cost structure, environmental attributes, and efficacy needed to generate net benefits for community-based tick control programs.

## 1. Introduction

Tickborne diseases are of increasing concern in the United States. Lyme disease and other tickborne diseases affect hundreds of thousands of people annually, with the highest risks for specific diseases concentrated in focal areas (Kugeler et al., 2021). This burden is evident in the upper midwestern states of Michigan, Minnesota, and Wisconsin. Minnesota and Wisconsin are both considered high incidence states for Lyme disease, while Michigan has focal areas of high incidence and evidence of recent geographic expansion of *Ixodes scapularis* (blacklegged tick), the primary vector of Lyme disease and several other tickborne diseases (Fleshman et al., 2022; Lantos et al., 2017; Rosenberg et al., 2018). The increase in reported tickborne disease cases in recent years is due to a multitude of complex factors, including but not limited to increased awareness and testing by healthcare providers, geographic expansion of vectors, and discovery of novel tickborne disease agents (Brett et al., 2014; Eisen and Eisen, 2018).

An important factor contributing to this increased burden has been the lack of access to or acceptance of effective tick control interventions. Tickborne disease prevention relies largely on personal protection methods, such as checking for ticks, showering after being outdoors, and using Environmental Protection Agency (EPA)-registered tick repellents or permethrin-treated clothing. Unfortunately, the effectiveness of these methods is somewhat limited by lack of proper or consistent use among those at risk of tick bites (Butler et al., 2016; Corapi et al., 2007; Hook et al., 2015; Niesobecki et al., 2019; Schwartz et al., 2022). For instance, the use of EPA-registered tick repellents is highly effective and safe, yet less than half of consumers in the upper Midwest report consistent use, citing common barriers like forgetting to use or bring repellent, concern about its safety, and a general dislike of the product (Beck et al., 2022). Furthermore, while tick checks are commonly recommended by public health officials and can be easily adopted by individuals, the small size of ticks can make it difficult for many people to detect them. And while the prospect of a new Lyme disease vaccine would have the potential to substantially reduce the number of Lyme disease cases that occur each year in the US, it would not diminish the burden of other tickborne diseases (Eisen, 2021; Gomes-Solecki et al., 2020; Shen et al., 2011).

Environmentally based tick control methods also exist. Currently available methods include landscaping (e.g., rock or mulch borders and leaf or brush removal), application of natural or synthetic pesticides to vegetation, or topical pesticide treatment of rodents and deer, which serve as reservoir hosts for ticks. While these methods allow for a more integrated approach to tick control, some lack sufficient data demonstrating their efficacy, many are not familiar options to the public, or in some cases, the products needed may only be available commercially through licensed operators. Additionally, concerns exist surrounding the safety of pesticides for the environment and wildlife, along with their feasibility in effectively treating large properties (Eisen, 2021; Eisen and Dolan, 2016; Eisen and Eisen, 2018; Hansen et al., 2025; Jordan and Schulze, 2020).

Property-based tick control methods can be limited because ticks and their hosts do not abide by property lines, meaning that the intended benefits of treating a single property could be impacted by the choice not to treat by a neighbor. One way to combat this issue would be to implement broad-scale, community-based tick control that would reduce ticks and tickborne diseases across entire neighborhoods of residential and recreational properties (Keesing et al., 2022; Schulze et al., 2023). At the community level, tick bite prevention and tick control methods could involve educational outreach, application of natural or synthetic pesticide to vegetation, treating rodents or deer with a pesticide, and/or landscaping efforts (e.g., maintaining trails and mowing frequently). While there is overlap in the available methods, the distinction we make here between individual property-based tick control and community-based tick control is in who makes the decision on what intervention is deployed (individual decision vs. individual decision made in collaboration with coordinated vector control program) and how it is funded (individually vs. contribution through taxes). Community level integrated pest management approaches like this are not commonly adopted for tick control, largely due to the lack of scientific data demonstrating efficacy in reducing human disease, a lack of acceptance and approval by the public, and the daunting task of getting community members to agree on a shared approach (Eisen and Eisen, 2018; Mader et al., 2021).

Willingness to adopt various tick control and tickborne disease prevention methods has been studied in different geographic regions of the US, but fewer studies have assessed the public’s willingness to pay for tick control methods (Beck et al., 2022; Bron et al., 2020; Butler et al., 2016; Gould et al., 2008; Hook et al., 2015; Niesobecki et al., 2019). We analyzed survey data from residents of Lyme disease-endemic counties of Michigan, Minnesota, and Wisconsin to assess their willingness to support and pay for tick control methods for residential properties and communities. These results will be informative for more in-depth willingness to pay studies in the Upper Midwest and elsewhere, but also stimulate research and discussions on effective, flexible, and creative community-supported approaches to reducing ticks and tickborne disease cases.

## 2. Materials and methods

We conducted a cross-sectional survey in the summer of 2019 in select counties of Michigan, Minnesota, and Wisconsin, with methods described previously (Beck et al., 2022). Briefly, a random sample of 39, 900 residential addresses was obtained from a marketing service company (Marketing Systems Group, Horsham, Pennsylvania, USA), targeting ‘high risk’ counties in each state, defined as those having a five-year average (2013–2017) Lyme disease incidence ≥10 cases per 100,000 persons per year (totaling 59 counties in Wisconsin, 49 counties in Minnesota, and 8 counties in Michigan). Respondents were asked to complete a web-based survey with the option to take the survey by phone upon request. All surveys were administered using Research Electronic Data Capture (REDCap) software (Vanderbilt University, Nashville, Tennessee, USA) hosted at the Minnesota Department of Health. The full survey consisted of five sections, with questions regarding perceived risk of tick bites, knowledge of tickborne diseases and prevention methods, willingness to participate in prevention behaviors, communication and outreach preferences, and demographics. For this analysis, we focused on the section on prevention and control (Section 3), which included specific questions regarding willingness to support and pay annually for various methods of property-based tick control (Appendix A). We also examined demographic characteristics that might influence behavior.

In this section of the survey, respondents were given two hypothetical situations, one where they were asked to consider different options for controlling ticks on their property, and another where they were asked about publicly funded community-level control. In both scenarios, they were instructed to imagine that the products used would not threaten human or animal health and would pose minimal risk to the environment. Additionally, they were told that the products used would reduce by half the probability that someone in their household would get a tickborne disease (for the private option) or reduce by half the number of people in the county that would get a tickborne disease (for the community option).

For the private option, respondents could indicate a preference for implementing a control method themselves, hiring a professional to do the work, or they could also indicate no interest in property-based control methods. Outcome variables included willingness to support and pay annually for residential property-based tick control methods: natural or synthetic pesticide application, devices that apply pesticides to rodents to kill ticks, or landscaping (e.g., hardscaping, rock or mulch borders, leaf or brush removal). For the community control option, outcomes included willingness to support and pay annually for any of five tick control methods in a county-wide program: natural or synthetic pesticide application, treating rodents with a pesticide, treating deer with a pesticide, or landscaping (e.g., maintaining trails, mowing frequently, and removing leaves and brush). They could also indicate no interest in community-based control methods. If respondents indicated support for any of the proposed methods, follow-up questions were asked to ascertain willingness to pay per year for each method, in increasing increments. Statistical analyses were conducted using Stata v. 17 (Stata Corp LLC, College Station, Texas, USA). We present results using ordered logit models and binary logit models. The study protocol was reviewed and approved by the Institutional Review Boards at the Minnesota Department of Health (IRB: #19–502) and the Centers for Disease Control and Prevention (IRB: #7217). Review boards at Michigan Department of Health and Human Services, Michigan State University, and the University of Wisconsin-Madison deferred to the Minnesota Department of Health’s authority.

## 3. Results

A complete description of the sample characteristics is available in Beck et al. (2022). In brief, a total of 2,836 respondents completed the survey and 2,713 surveys (96 %) met criteria to be included in the analysis. Most reported that they were likely to encounter ticks recreationally around their community (91 %) or around home (75 %), and although the percentages varied by disease, the majority of people felt that tickborne diseases were very serious. While only 25 % of respondents thought that tickborne diseases were common in their community, 72 % felt that either they or someone in their household was at least slightly likely to get sick in the next year.

When presented with the hypothetical scenario on tick control, 2,160 respondents (79 %) were interested in property-level control, 495 respondents were not, and 58 did not respond. Of those who were willing to perform some form of tick control on their property, 1,719 (79 %) indicated a preference for applying over-the-counter tick control products to or landscaping their property themselves while 20 % (441 respondents) preferred hiring a pest control company to perform control activities.

### Willingness to Pay for Residential Property-Based Tick Control Methods

#### Willingness to Pay for Methods Involving Self-Application

**Fig. 1.**
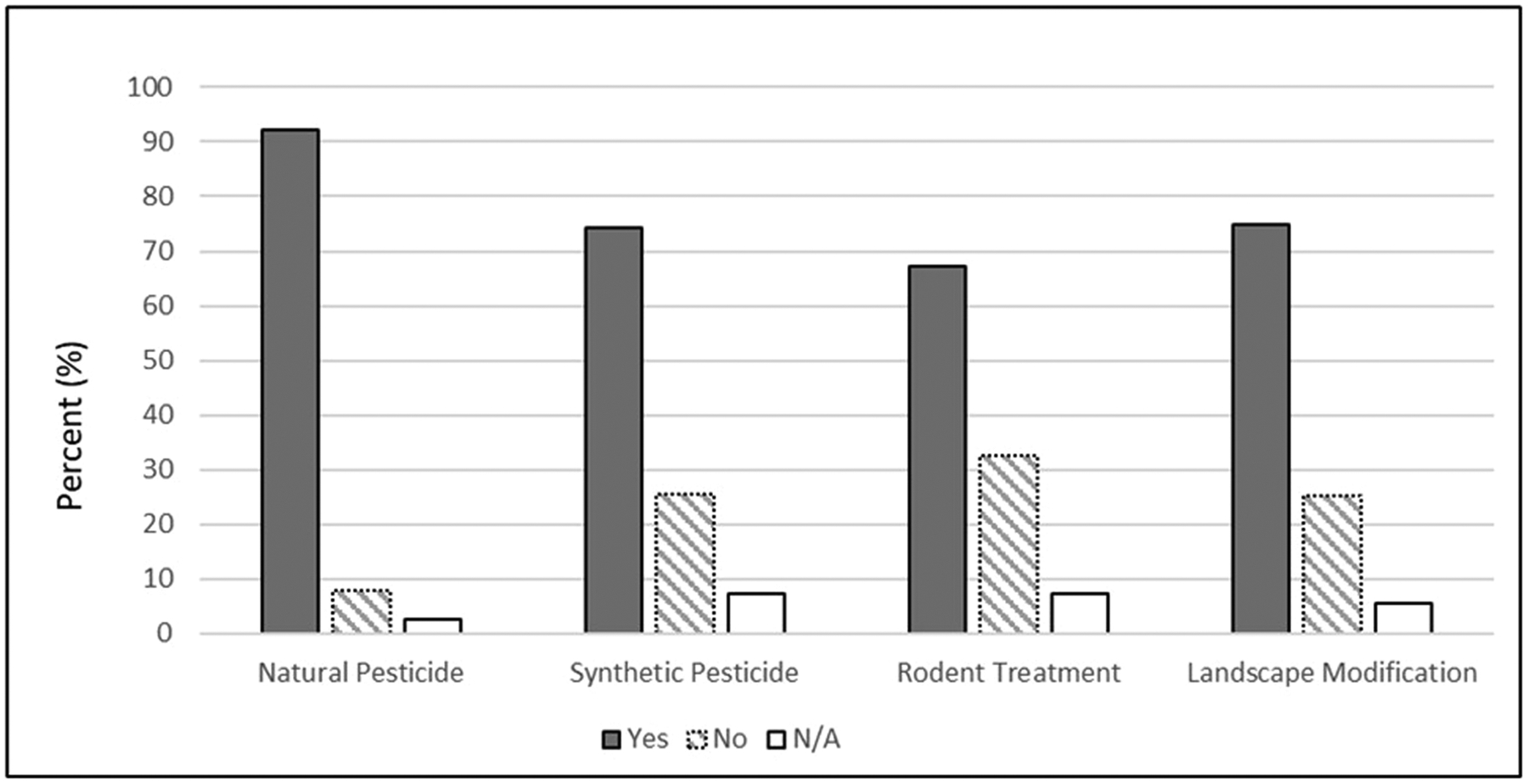
Percent of survey participants willing to pay for self-application of tick control methods, including natural or synthetic pesticide, applying pesticide to rodents, and landscaping. N/A represents the respondents that were not interested in any sort of property level tick control. Reported totals include the sum of participants in favor of a control method across all states (MI, MN, WI).

Of 1,719 respondents who chose to implement control themselves, more (90 %) were willing to pay for natural than for synthetic (70 %) or rodent-targeted (63 %) pesticides, or for landscaping (71 %). Among those willing to pay at least something for pesticide application (Fig. 1), more than 80 % (depending on method) were willing to pay up to $100/year. Nearly 30 % of those willing to pay for landscaping, however, were willing to pay more than $100/year (Figure B.1). Household characteristics of those willing to pay for and perform residential property-based tick control methods themselves were similar across all four tick control options (natural pesticide, synthetic pesticide, treating rodents with pesticide, landscaping).

For each control method, respondents selecting self-application indicated which of six ranges ($0, $1-$49, $50-$99, $100-$299, $300-$499, >$500) best described the amount they were willing to pay. Responses were analyzed using an ordered logit model to predict the probability of respondents selecting each of the six ranges, conditional on household characteristics. Model parameters indicate whether a characteristic makes a household more or less likely to select a higher willingness to pay category. Estimates for each of the four control methods are shown in Table B.1, and the number of respondents in each household type is shown in Table C.1. Respondents having a high income (defined as an annual household income before taxes of more than $100,000) and those with a high perceived prevalence of disease (defined as people who said that tickborne diseases were very or extremely common) were more likely to select higher willingness to pay ranges for all the control options. The willingness to pay ranges for synthetic pesticides and devices to apply pesticides to rodents were higher among males than females, while respondents with a high perceived seriousness of disease (defined as people who said that tickborne disease is either very or extremely serious) were more likely to choose a higher willingness to pay category for both natural and synthetic pesticide. Those with a high likelihood of disease (defined as people who said that they or someone in the household was very or extremely likely to get a tickborne disease) would pay more for natural and synthetic pesticides and rodent-targeted pesticides, but respondents reporting a past diagnosis would pay more for natural pesticides than other options. Additional covariates, such as age over 50, less than a college-level education, and state of residence were not individually statistically significant predictors of choices.

Ordered logit estimates were used to predict the annual willingness to pay for each treatment option as a weighted sum of the midpoints of the payment ranges ($24, $74.5, $199.5, $399.5, $500), where the weights are the predicted probabilities corresponding to each payment range. Predictions were made conditional on values for household characteristics. The sample average respondent was willing to pay $78 (95 % CI: $74, $83) and $61 (95 % CI: $57, $64) annually for the most preferred control methods of landscaping and natural pesticide application, respectively. In comparison, they were willing to pay $45 (95 % CI: $42, $48) and $37 (95 % CI: $35, $40) annually for the least preferred control methods of synthetic pesticide and rodent treatment devices, respectively (Table 1). When characteristics of households such as income, education, and perceived risk of disease were considered, the largest variability in payment amounts was for natural pesticide applications ($35 to $129, with non-overlapping confidence intervals). For all control options, those households having a college education, high income, past tickborne disease diagnosis, and high perceived prevalence, seriousness, and likelihood of disease were willing to pay the most, though in many cases confidence intervals overlapped.

#### Willingness to Pay for Methods Involving Commercial Application

**Fig. 2.**
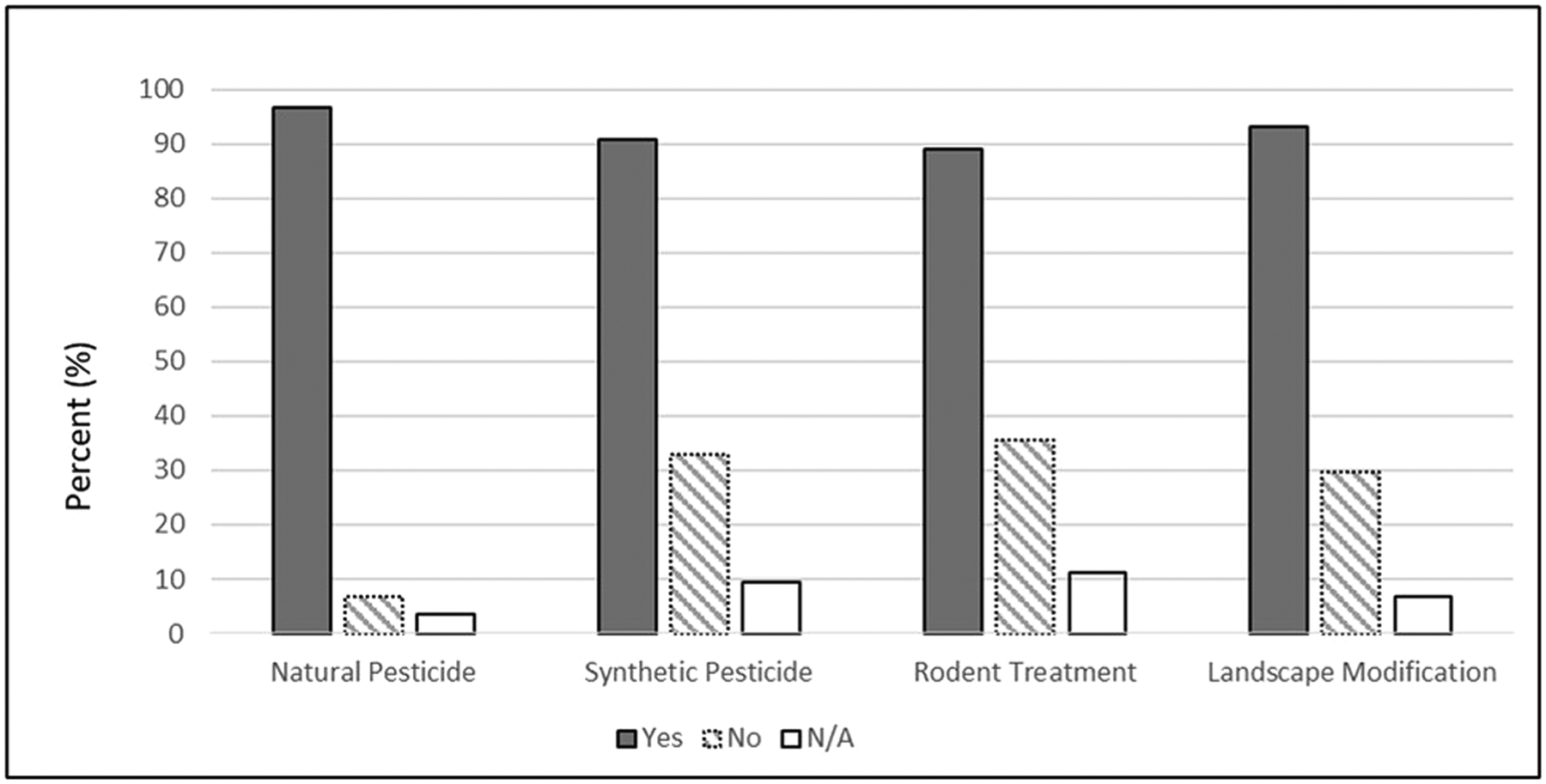
Percent of survey participants willing to pay for commercial application of tick control methods, including natural or synthetic pesticide, applying pesticide to rodents, and landscaping. N/A represents the respondents that were not interested in any sort of property level tick control. Reported totals include the sum of participants in favor of a control method across all states (MI, MN, WI).

Of 441 respondents who reported a preference for hiring a pest control company rather than performing control themselves, natural pesticide application was the most popular, with 94 % willing to pay at some amount (Fig. 2). Roughly 75 % were willing to pay for each of the other three options (synthetic pesticide, landscaping, rodent treatment devices). Responses were again analyzed using an ordered logit model (Table B.2) and the same weighted sum approach to estimate annual willingness to pay. The six ranges for professional application options were $0, $1-$99, $100-$299, $300-$499, $500-$999, and > $1000, with weighted average midpoints of $50, $99.5, $399.5, $749.5, and $1000. When compared to the results for self-application, the findings for commercial application were similar. The sample average respondent was willing to pay the most for commercial landscaping ($155/year) and application of natural pesticides ($112/year). In addition, while some also indicated a willingness to pay for synthetic pesticides and rodent treatment devices, they were not willing to spend as much money ($90 and $72 annually, respectively) on these options (Table 2). When household characteristics were considered, the largest variability in payment amounts was again for natural pesticide applications ($87 to $345 annually). The characteristics of households willing to pay the most were the same as for those that preferred to implement control themselves. Only two household characteristics were consistently associated with willingness to pay for commercial application for any of the control options: having a high income and a high perceived likelihood of disease (Table B.2). In general, the smaller sample size of those choosing commercial application led to less precise coefficient estimates for the ordered logit and wider confidence intervals for willingness to pay.

### 3.1. Community-based methods

#### Willingness to Pay for Community-Based Tick Control Methods

Nearly all (2,625, or 97 %) respondents indicated interest in at least one of the five program options (natural pesticide, synthetic pesticide, treating rodents with pesticide, treating deer with pesticide, landscaping) that could be used for community tick control and received a follow-up question regarding how much they would be willing to pay per year as a household surcharge for a county program. Approximately 80 % of those respondents said ‘yes’ for the initial $10 option and were asked if they would be willing to pay $30. Among those who received the $30 option, 60 % said ‘yes’ and were asked about paying $50 for the program; 58 % said ‘yes.’ In total, with both the initial and follow-up questions, there were 5,973 binary responses to the $10, $30, and $50 payment levels. Outcomes were stacked, and binary logit models that predict the probability of a ‘yes’ answer based on the payment level and household characteristics were estimated. Estimates for five specifications are shown in Table B.3, expressed as parameters. Using our preferred specification, we found that the sample average household was willing to pay $52/year (95 % CI: $48, $56) for a community-based program where they live. When household characteristics were considered, the range in estimated payment amounts was between $28 (95 % CI: $20, $35) and $89 (95 % CI: $78, $101) annually; households having some college education, high income, past tickborne disease diagnosis, and high perceived prevalence, seriousness, and likelihood of disease were willing to pay the most (Table 3).

**Fig. 3.**
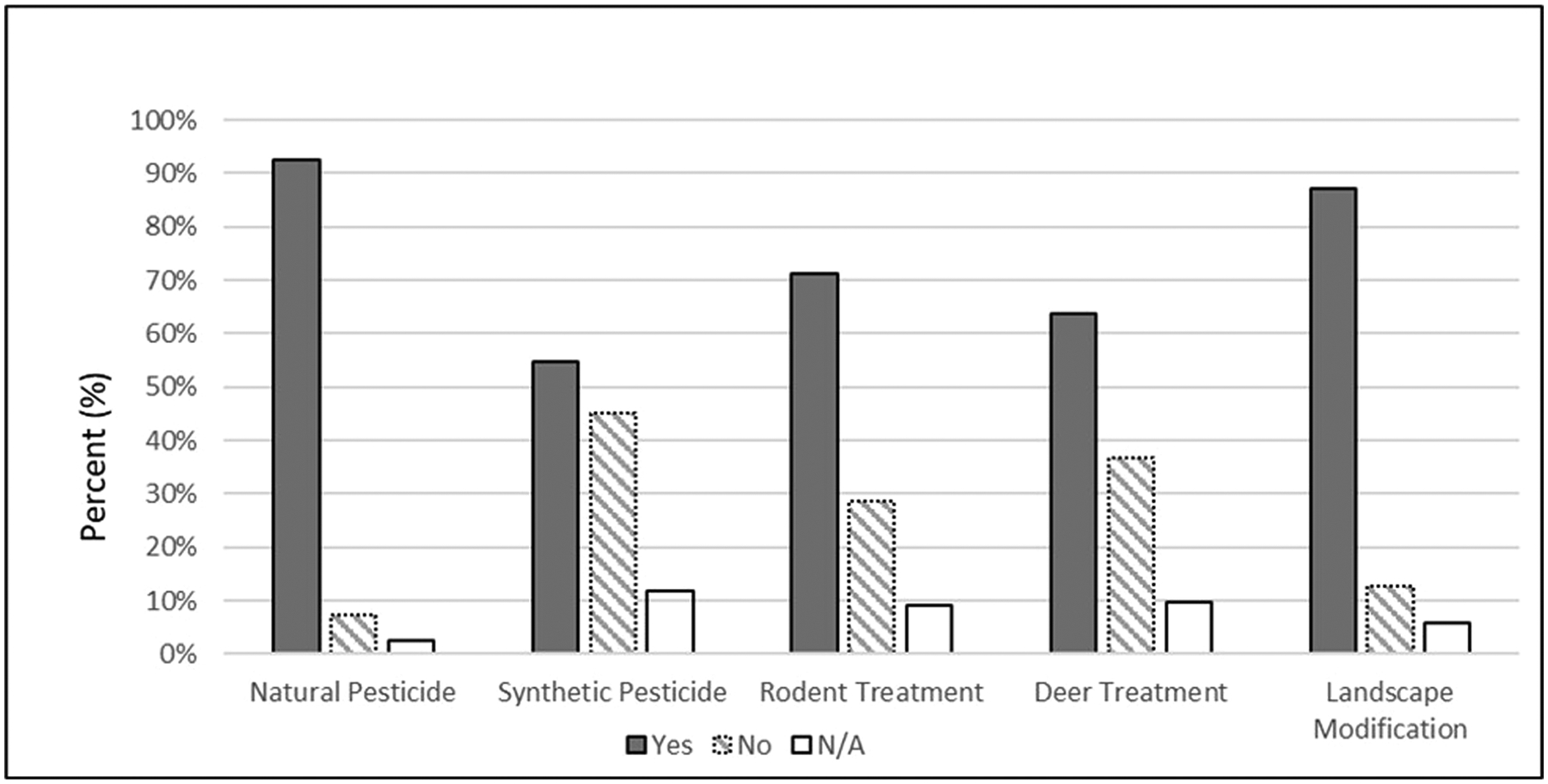
Percent of survey participants willing to pay for community-level tick control methods, including natural or synthetic pesticide, applying pesticide to rodents or deer, and landscaping. N/A represents the respondents that were not interested in any sort of property level tick control. Reported totals include the sum of participants in favor of a control method across all states (MI, MN, WI).

Similar to the preferences in method that were found for private control options, spraying with a natural pesticide and landscaping were the most popular, with 93 % of respondents in favor of spraying and 87 % supporting landscape changes such as maintaining trails, mowing frequently, and removing leaves and brush. Treating rodents (71 %) and treating deer (64 %) with pesticide were also well supported, while spraying with a synthetic pesticide was less popular, with only 55 % of respondents supporting it (Fig. 3).

## 4. Discussion

This study of residents from high-incidence counties for Lyme disease in Michigan, Minnesota, and Wisconsin indicates an overall moderate willingness among respondents to support and pay for a variety of tick control options for their individual property as well as the broader community. The hypothetical scenarios used to evaluate willingness asked respondents to consider both private and community options. Regardless of whether respondents preferred to do it themselves or hire someone to conduct tick control, the majority were willing to pay at least some amount for tick control, with a preference for natural pesticides over synthetic pesticides, rodent-targeted approaches, and landscaping. However, of those willing to pay at least some amount, a larger share of respondents were willing to pay more for landscaping than for other control methods. When considering community tick control efforts, most respondents similarly favored application of natural pesticides.

Previous studies reported a maximum willingness to pay of $100 annually for residential property-based tick control methods (Gould et al., 2008; Niesobecki et al., 2019; Niesobecki et al., 2022; Schotthoefer et al., 2020). In this study, the respondents’ willingness to pay higher amounts varied by control method and by applicator. Participants were willing to pay approximately twice as much for tick control options – up to $155 on average for landscaping and $112 for application of natural products – when performed by a professional. Characteristics associated with respondents’ willingness to pay for tick control were similar for individual properties and publicly funded community options. In general, wealthier households with higher levels of education, in areas with high perceived prevalence and likelihood of disease had a higher willingness to pay. Given that all respondents lived in areas with a high reported rate of Lyme disease, these findings suggest that demand for tick control may generally be higher when people perceive the prevalence, seriousness, and likelihood of health risks to be higher. Broader demand for tick control could potentially increase with public health education designed to align risk perception with actual risk.

In this study, we found that people were willing to pay an average of $52 annually in taxes annually to support a community tick control program where they live. There is scant literature regarding willingness to pay for publicly funded community-based tick control, though some comparisons can be drawn from similar work with mosquito control programs. Many of these programs have existed for decades, are publicly funded, and use integrated pest management approaches. Surveys in Florida and Arizona have estimated that roughly half of survey respondents were willing to pay at least $100 per year for mosquito control (Dickinson et al., 2016). Another survey in Wisconsin showed that over 80 % of respondents were willing to pay at least $10, and 25–33 % were willing to pay the maximum response of $200 per year for mosquito control (Dickinson and Paskewitz, 2012). Implementing a community-wide publicly funded tick control program may be more difficult, as willingness to pay for mosquito control may largely be driven by concerns over nuisance biting rather than disease prevention.

It is not appropriate, however, to compare results for private and community options, given that these two options were asked as separate questions and have different interpretations. Private demand is about a single household, and the purchased service (tick control) is only about controlling ticks on that individual property. If broadly and effectively deployed, a publicly funded community-based program is actually nonrival; all households in the community simultaneously benefit from the program, creating what economists refer to as a local public good. While it is true that the willingness to pay in a community for a local public good is the summation of households’ individual willingness to pay, it is not always that simple. Having an effective and accepted community program would likely decrease the demand for private control and may even reduce the practice or use of personal protection options; in this analysis, though, we chose to characterize private demand as conditional on there being no community-based program and that use of personal protection would be unaffected.

The original KAB study by Beck et al. (2022) reported that only 13 % of participants had ever treated their property (including vacation homes and cabins) with a pesticide for the purposes of killing ticks. This low percentage stands in stark contrast to the more than 60 % of participants who reported being willing to pay at least some amount for any of the tick control methods for their individual properties. The large gap between those who have used acaricide on their property and those who are willing to do so may present an opportunity for public health outreach and development of national- or state-level guidance for residential tick control, perhaps especially since most respondents in our study preferred to treat property themselves versus hiring a professional. Nevertheless, it is clear from both this and past efforts that there is a preference for pesticide products that are labeled as “natural” (Niesobecki et al., 2019; Niesobecki et al., 2022). Since this nomenclature may be used on labels of products with a wide range of active ingredients and, concordantly, a wide range of expected efficacies to kill ticks, any guidance should include product-specific efficacy, use, and safety information. We saw a general willingness to pay larger amounts for landscaping, which may reduce tick habitat, may minimize the need for pesticides (of all kinds), and have lasting effects across multiple tick seasons. Although detailed guidance regarding the most effective and lasting tick control approaches may be beneficial as a resource for the general public and landscaping professionals, more research is needed on efficacy before it can be developed. One survey among publicly funded vector control program staff where *Ixodes*-transmitted pathogens are common, including in the Upper Midwest, showed some willingness to expand services to provide tick suppression guidance to the public, if funding and personnel barriers could be overcome (Burtis et al., 2024).

These results must, however, be contextualized within the setting of currently available tick control options. Notably, many of the methods asked about, such as residential yard-based acaricide applications (Hinckley et al., 2016), rodent-targeted bait boxes (Hinckley et al., 2021), and neighborhood-based approaches (Keesing et al., 2022; Ostfeld et al., 2023) have not been shown to reduce risk for tick encounters or tickborne disease. Further, a recent survey among private pest control operators revealed that natural pesticides, landscape management, and host-targeted devices are not commonly offered (Jordan et al., 2024). Another survey among publicly funded vector control programs in Lyme disease endemic areas in the Northeastern U.S. found that only 11 % conduct tick control activities in their communities, with low willingness to expand services to private property, even if funding, training, and resources were not a limitation (Burtis et al., 2024). So, while our survey questions presupposed efficacy and availability, there exists a gap between respondents’ stated preferences for tick control methods and the actual feasibility, effectiveness, and cost of currently available tick control. As incidence and range of tickborne diseases continues to increase in the U.S., this gap highlights the continuing need for promotion of personal protective measures and utility of developing novel methods, such as vaccines.

There are a number of potential limitations of this study. Although people indicated a willingness to pay for control, and every attempt was made to provide reasonable cost options, the questions in this survey may not have provided cost choices that were realistic or aligned with current market values in the areas where participants live. If average costs of control are actually higher than the willingness to pay from this study, as indicated in other recent studies (Jordan and Schulze, 2020; Schulze et al., 2023) it may help explain the relatively low rates of current property control reported in Beck et al. (2022). It is also possible that our results were affected by selection bias, as respondents who took the survey may be more likely to be interested in tick control than the general population, potentially overestimating true demand and willingness to pay. Respondents in our sample also reported an overall higher level of education than what is shown in state-level demographic data (Beck et al., 2022), which could lead to an overestimate of the effect of education on willingness to pay. Finally, the generalizability of these findings is limited to English speakers living in regions of high incidence for Lyme disease in the Midwest.

The results of this study suggest there is moderate demand for tick control efforts at both the individual property level and for local, publicly funded, community-based programs. Our per-household willingness to pay (and the implied total willingness to pay for community programs) estimates provide a starting point for assessing the cost structure and efficacy to generate net benefits from a public finance perspective. Ultimately, these results set the stage for future studies regarding willingness to pay and cost-benefit analysis for community control efforts, provide insights regarding product use and preferences, and highlight opportunities for improved guidance and education.

## Supplementary Material

Supplementary Material

Supplementary material associated with this article can be found, in the online version, at doi:10.1016/j.ttbdis.2025.102531.

## Figures and Tables

**Table 1:** Participant household characteristics and willingness to pay (WTP) annually for self-applied tick control methods.

	Household Characteristic						Average Predicted WTP Amount by Method ($, 95 % CI)			
Household Type	College	High income^1^	High perceived prevalence of disease^2^	High perceived seriousness of disease^3^	High perceived likelihood of disease^4^	Past diagnosis of disease	Spraying natural pesticide	Spraying synthetic pesticide	Applying pesticide to rodents	Landscape modification
Sample	0.84	0.18	0.33	0.91	0.06	0.19	$61 (57, 64)	$45 (42, 48)	$37 (35, 40)	$78 (74, 83)
1							$35 (28, 41)	$29 (22, 36)	$25 (18, 31)	$61 (47, 75)
2	×						$40 (33, 46)	$30 (24, 36)	$27 (22, 33)	$79 (64, 93)
3	×	×					$55 (45, 65)	$42 (33, 51)	$38 (30, 47)	$106 (86, 127)
4	×	×	×				$65 (52, 79)	$55 (42, 67)	$47 (36, 58)	$120 (96, 144)
5	×	×	×	×			$87 (76, 99)	$69 (59, 79)	$54 (46, 62)	$111 (95, 127)
6	×	×	×	×	×		$107 (86, 129)	$91 (71, 112)	$72 (56, 89)	$119 (92, 145)
7	×	×	×	×	×	×	$129 (105, 153)	$87 (68, 107)	$78 (61, 95)	$125 (98, 152)
8			×	×	×	×	$85 (67, 103)	$64 (49, 79)	$55 (41, 68)	$74 (54, 93)
9	×		×	×			$64 (58, 70)	$51 (45, 56)	$40 (35, 45)	$82 (73, 92)
10	×					×	$49 (39, 58)	$28 (21, 36)	$30 (23, 38)	$83 (65, 101)

Values in this table are based on the four sets of ordered logit estimates in Table B.1 and represent the annual willingness to pay amount for different types of households for the different control methods. Counts of respondents in each sample type are provided in Table C.1. Ninety-five percent confidence intervals calculated using the delta method are shown in parentheses. The willingness to pay estimates are based on a weighted sum of the midpoints of the payment ranges presented in the survey, where the weights are the predicted ordered logit probabilities corresponding to each payment range. That is:

$$WTP=\hat{pr_{2}}\times 24+\hat{pr_{3}}\times 74.5+\hat{pr_{4}}\times 199.5+\hat{pr_{5}}\times 399.5+\hat{pr_{6}}\times 500.$$

The probabilities were constructed based on the specific household type, with covariate values substituted into the probability formula. For the sample row, the displayed sample averages are substituted into the formula. We also used sample proportions for over 50 years old (0.642), MI resident (0.331), and WI resident (0.342). For types 1–10, the covariate values substituted into the probability formula were 0 or 1, with an ‘x’ in the column meaning that characteristic was set to 1. Sample proportions were again used for over 50 years old, MI resident, and WI resident. For example, a type 3 household has a college educated and high-income respondent who does not perceive high disease prevalence, seriousness, or likelihood and has not been previously diagnosed.

1 High income is defined as an annual household income before taxes of more than $100,000

2 High perceived prevalence of disease is defined as people who said that tickborne diseases were very or extremely common in the community where they live

3 High perceived seriousness is defined as people who said that tickborne disease is either is very or extremely serious

4 High perceived likelihood of disease is defined as people who said that they or someone in the household was very or extremely likely to get a tickborne disease

**Table 2:** Participant household characteristics and willingness to pay (WTP) annually for commercially applied tick control methods.

	Household Characteristic						Average Predicted WTP Amount by Method ($, 95 % CI)			
Household Type	College	High income^1^	High perceived prevalence of disease^2^	High perceived seriousness of disease^3^	High perceived likelihood of disease^4^	Past diagnosis of disease	Spraying natural pesticide	Spraying synthetic pesticide	Applying pesticide to rodents	Landscape modification
Sample	0.85	0.27	0.32	0.90	0.07	0.16	$112 (98, 126)	$90 (76, 104)	$72 (61, 83)	$155 (134, 176)
1							$87 (59, 114)	$58 (32, 84)	$66 (38, 93)	$118 (65, 171)
2	×						$97 (70, 125)	$74 (47, 101)	$85 (55, 114))	$169 (111, 226)
3	×	×					$139 (92, 186)	$111 (67, 154)	$113 (69, 156)	$257 (168, 347)
4	×	×	×				$166 (99, 234)	$102 (55, 150)	$130 (71, 187)	$311 (192, 430)
5	×	×	×	×			$156 (117, 196)	$118 (83, 154)	$100 (70, 129)	$243 (180, 306)
6	×	×	×	×	×		$301 (168, 434))	$205 (98, 311)	$126 (61, 192)	$276 (137, 415)
7	×	×	×	×	×	×	$345 (211, 478)	$170 (89, 251)	$112 (69, 165)	$309 (178, 441)
8			×	×	×	×	$210 (117, 302)	$92 (43, 142)	$65 (31, 99)	$148 (64, 232)
9	×		×	×			$109 (86, 131)	$79 (58, 101)	$75 (55, 94)	$158 (117, 199)
10	×					×	$111 (70, 153)	$60 (29, 91)	$74 (40, 108)	$193 (110, 275)

Values in this table are based on the four sets of ordered logit estimates in Table B.2 and represent the annual willingness to pay amount for different types of households for the different control methods. Counts of respondents in each sample type are provided in Table C.1. Ninety-five percent confidence intervals calculated using the delta method are shown in parentheses. The willingness to pay estimates are based on a weighted sum of the midpoints of the payment ranges presented in the survey, where the weights are the predicted ordered logit probabilities corresponding to each payment range. That is:

$$WTP=\hat{pr_{2}}\times 50+\hat{pr_{3}}\times 99.5+\hat{pr_{4}}\times 399.5+\hat{pr_{5}}\times 749.5+\hat{pr_{6}}\times 1000.$$

The probabilities were constructed based on the specific household type, with covariate values substituted into the probability formula. For the sample row, the displayed sample averages were substituted into the formula. We also used sample proportions for over 50 years old (0.567), MI resident (0.293), and WI resident (0.340). For types 1–10, the covariate values substituted into the probability formula were 0 or 1, with an ‘x’ in the column meaning that characteristic is set to 1. Sample proportions were again used for over 50 years old, MI resident, and WI resident. For example, a type 3 household has a college educated and high-income respondent who does not perceive high disease prevalence, seriousness, or likelihood and has not been previously diagnosed.

1 High income is defined as an annual household income before taxes of more than $100,000

2 High perceived prevalence of disease is defined as people who said that tickborne diseases were very or extremely common in the community where they live

3 High perceived seriousness is defined as people who said that tickborne disease is either is very or extremely serious

4 High perceived likelihood of disease is defined as people who said that they or someone in the household was very or extremely likely to get a tickborne disease

**Table 3:** Participant household characteristics and willingness to pay (WTP) annually for a community-level tick control program.

	Household Characteristic						
Household Type	College	High income^1^	High perceived prevalence of disease^2^	High perceived seriousness of disease^3^	High perceived likelihood of disease^4^	Past diagnosis of disease	Predicted Annual Average WTP ($, 95 % CI)
Sample	0.84	0.20	0.32	0.90	0.06	0.19	$52 (48, 56)
1							$28 (20, 35)
2	×						$42 (35, 49)
3	×	×					$61 (52, 69)
4	×	×	×				$66 (57, 75)
5	×	×	×	×			$72 (65, 79)
6	×	×	×	×	×		$84 (72, 96)
7	×	×	×	×	×	×	$89 (78, 101)
8			×	×	×	×	$56 (46, 67)
9	×		×	×			$53 (48, 58)
10	×					×	$47 (39, 56)

Values in this table were derived from the binary logit estimates from Model E in Table B.3. They represent the annual household-level willingness to pay for a community tick control program. Counts of respondents in each sample type are provided in Table C.1. Ninety-five percent confidence intervals calculated using the delta method are shown in parentheses. Willingness to pay was calculated according to

$$WTP=-\frac{\sum \gamma_{k}X_{k}}{\beta },$$

where *X*_*k*_ is a non-price variable (e.g., past diagnosis), *g*_*k*_ is the parameter estimate on *X*_*k*_, and *b* is the parameter estimate on price. WTP varies continuously and is not bound by the range of values for price. Estimates of willingness to pay were constructed for specific household types, with covariate values substituted into the formula. For the sample row, the displayed sample averages are entered into the formula. For types 1–10, the covariate values substituted into the formula were 0 or 1, with an ‘x’ in the column meaning the characteristic was set to 1. For example, a type 3 household has a college educated and high-income respondent who does not perceive high disease prevalence, seriousness, or likelihood and has not been previously diagnosed.

1 High income is defined as an annual household income before taxes of more than $100,000

2 High perceived prevalence of disease is defined as people who said that tickborne diseases were very or extremely common in the community where they live

3 High perceived seriousness is defined as people who said that tickborne disease is either is very or extremely serious

4 High perceived likelihood of disease is defined as people who said that they or someone in the household was very or extremely likely to get a tickborne disease

## Data Availability

Survey respondents were assured that the raw data from this survey would remain confidential, and in only summary data would be published or shared. Raw data are not available.

## Data availability

The data that has been used is confidential.
