# Supplementary material for: Willingness to pay for residential property-based and community-based tick control methods in Lyme disease-endemic areas of the Upper Midwest, United States
Source: Ticks Tick Borne Dis. Author manuscript; Available in PMC 2026 Aug 6. (PMC12435866; doi:10.1016/j.ttbdis.2025.102531)
Supplement: Supplementary file 1 [file NIHMS2109108-supplement-Supplementary_Material.docx]

**APPENDIX A:**

Prevention and control questions from the 2019 Upper Midwest Tick-borne Disease Prevention Survey.

**APPENDIX B:**

**Figure B.1**. Percent of survey participants willing to pay annually for residential property-based tick control methods by amount and method.

 This figure compares what survey respondents were willing to pay, per year, to either self-apply control methods or landscape modification (left panel) or to obtain these services from a commercial provider (right panel). The cost ranges for each are slightly different, but results were similar across both groups. Fewer respondents were willing to pay for any method as prices increased, although a willingness to pay for landscape modification remained relatively high in comparison to other methods.

**Table B.1:** Willingness of participants to pay annually for self-application of tick control methods.

|  | **Spraying Natural Pesticide (*n*=1,678)** | | **Spraying Synthetic Pesticide**  **(*n*=1,604)** | | **Applying Pesticide to Rodents**  **(*n*=1,604)** | | **Landscape Modification (*n*=1,631)** | |
| --- | --- | --- | --- | --- | --- | --- | --- | --- |
| **Variable** | **Odds Ratio** | **95% CI** | **Odds Ratio** | **95% CI** | **Odds Ratio** | **95% CI** | **Odds Ratio** | **95% CI** |
| Age > 50 | 1.02 | (0.84, 1.24) | 1.04 | (0.86, 1.26) | 1.11 | (0.92, 1.35) | 0.87 | (0.72, 1.04) |
| Elementary/Middle/HS | 0.77 | (0.59, 0.99) | 0.95 | (0.74, 1.23) | 0.85 | (0.65, 1.11) | 0.66 | (0.52, 0.85) |
| High Income | 1.93^*^ | (1.51, 2.47) | 1.76^*^ | (1.38, 2.24) | 1.70^*^ | (1.34, 2.16) | 1.70^*^ | (1.33, 2.17) |
| Male | 0.90 | (0.75, 1.09) | 1.59^*^ | (1.32, 1.92) | 1.24^*^ | (1.03, 1.50) | 0.95 | (0.80, 1.14) |
| Perceived prevalence | 1.43^*^ | (1.16, 1.77) | 1.56^*^ | (1.27, 1.92) | 1.41^*^ | (1.15, 1.74) | 1.26^*^ | (1.03, 1.54) |
| Perceived seriousness | 1.84^*^ | (1.32, 2.57) | 1.53^*^ | (1.12, 2.10) | 1.30 | (0.95, 1.80) | 0.86 | (0.64, 1.17) |
| Perceived likelihood | 1.57^*^ | (1.05, 2.34) | 1.76^*^ | (1.17, 2.65) | 1.74^*^ | (1.16, 2.63) | 1.14 | (0.77, 1.70) |
| Past diagnosis | 1.52^*^ | (1.19, 1.95) | 0.92 | (0.71, 1.18) | 1.17 | (0.91, 1.49) | 1.10 | (0.86, 1.40) |
| MI | 1.12 | (0.90, 1.41) | 0.98 | (0.78, 1.23) | 0.80 | (0.64, 1.00) | 0.93 | (0.75, 1.16) |
| WI | 0.91 | (0.73, 1.14) | 0.81 | (0.65, 1.01) | 0.79^*^ | (0.64, 0.99) | 0.83 | (0.67, 1.03) |
| *Cut-off parameters* |  |  |  |  |  |  |  |  |
| Cut1 | -1.71 | (-2.11, -1.32) | -0.33 | (-0.69, 0.03) | -0.26 | (-0.62, 0.11) | -1.32 | (-1.68, -0.97) |
| Cut2 | 1.18 | (0.8, 1.57) | 1.63 | (1.26, 2.01) | 1.56 | (1.19, 1.93) | -0.08 | (-0.43, 0.27) |
| Cut3 | 2.92 | (2.52, 3.33) | 3.33 | (2.92, 3.74) | 3.38 | (2.96, 3.8) | 1.07 | (0.72, 1.42) |
| Cut4 | 5.02 | (4.48, 5.56) | 5.42 | (4.82, 6.02) | 5.65 | (4.93, 6.37) | 2.97 | (2.55, 3.38) |
| Cut5 | 6.29 | (5.50, 7.09) | 6.47 | (5.59, 7.35) | 6.34 | (5.39, 7.29) | 4.14 | (3.61, 4.68) |

Estimates are from ordered logit models for each of the four control options. Ordinal categories are $0 per year, $1-$49 per year, $50-$99 per year, $100-$299 per year, $300-$499 per year, and more than $500 per year. The models predict the probability of choice for each respondent for each of the six cost range categories. Significant odds ratios are marked with an asterisk. Odds ratios larger than one are associated with household characteristics that increase willingness to pay. For example, high income households have higher WTP for all control methods, relative to non-high income households.

Minnesota (MN) is reference state for all state specifications. Reference groups for the other variables, are, in order of appearance: age ≤ 50, college education, low income, female, low perceived prevalence, low perceived seriousness, low perceived likelihood of illness, and no past diagnosis.

**Table B.2:** Willingness of participants to pay annually for commercial application of tick control methods.

|  | **Natural Pesticide**  **(*n*=427)** | | **Synthetic Pesticide (*n*=410)** | | **Applying Pesticide to Rodents**  **(*n*=405)** | | **Landscape Modification (*n*=418)** | |
| --- | --- | --- | --- | --- | --- | --- | --- | --- |
| **Variable** | **Odds Ratio** | **95% CI** | **Odds Ratio** | **95% CI** | **Odds Ratio** | **95% CI** | **Odds Ratio** | **95% CI** |
| Age > 50 | 1.10 | (0.76, 1.59) | 1.13 | (0.78, 1.62) | 1.04 | (0.72, 1.50) | 0.86 | (0.61, 1.23) |
| Elementary/Middle/HS | 0.77 | (0.45, 1.31) | 0.67 | (0.4, 1.14) | 0.64 | (0.37, 1.09) | 0.57^*^ | (0.34, 0.96) |
| High Income | 2.13^*^ | (1.41, 3.22) | 1.98^*^ | (1.3, 3.01) | 1.69^*^ | (1.12, 2.57) | 2.08^*^ | (1.38, 3.12) |
| Male | 0.83 | (0.55, 1.25) | 0.94 | (0.63, 1.42) | 1.11 | (0.73, 1.67) | 0.88 | (0.59, 1.3) |
| Perceived prevalence | 1.43 | (0.93, 2.20) | 0.87 | (0.56, 1.34) | 1.31 | (0.85, 2.03) | 1.44 | (0.95, 2.18) |
| Perceived seriousness | 0.88 | (0.48, 1.62) | 1.29 | (0.72, 2.32) | 0.61 | (0.33, 1.12) | 0.63 | (0.36, 1.11) |
| Perceived likelihood | 3.73^*^ | (1.55, 8.98) | 2.68^*^ | (1.09, 6.61) | 1.56 | (0.64, 3.8) | 1.26 | (0.52, 3.05) |
| Past diagnosis | 1.34 | (0.78, 2.29) | 0.71 | (0.41, 1.26) | 0.79 | (0.45, 1.39) | 1.25 | (0.74, 2.12) |
| MI | 1.16 | (0.74, 1.83) | 0.89 | (0.57, 1.39) | 0.73 | (0.46, 1.14) | 1.25 | (0.81, 1.94) |
| WI | 1.10 | (0.71, 1.70) | 0.85 | (0.55, 1.31) | 0.96 | (0.62, 1.50) | 1.16 | (0.76, 1.77) |
| *Cut-off parameters* |  |  |  |  |  |  |  |  |
| Cut1 | -2.41 | (-3.12, -1.69) | -0.78 | (-1.41,- 0.15) | -1.29 | (-1.96, -0.63) | -1.33 | (-1.95, -0.72) |
| Cut2 | 0.39 | (-0.26, 1.04) | 0.80 | (0.17, 1.43) | 0.48 | (-0.17, 1.14) | -0.29 | (-0.89, 0.31) |
| Cut3 | 2.62 | (1.92, 3.33) | 2.69 | (1.98, 3.39) | 2.58 | (1.83, 3.33) | 1.21 | (0.59, 1.83) |
| Cut4 | 3.97 | (3.13, 4.81) | 4.31 | (3.37, 5.25) | 4.11 | (3.04, 5.19) | 2.49 | (1.81, 3.17) |
| Cut5 | 5.03 | (3.94, 6.13) | 4.6 | (3.59, 5.62) | 4.63 | (3.34, 5.92) | 3.79 | (2.92, 4.67) |

Estimates are from ordered logit models for each of the four control options. Ordinal categories are $0 per year, $1-$99 per year, $100-$299 per year, $300-$499 per year, $500-$999 per year, and more than $1,000 per year. The models predict the probability of choice for each respondent for each of the six cost range categories. Significant odds ratios are marked with an asterisk. Odds ratios larger than one are associated with household characteristics that increase willingness to pay. For example, high income households have higher WTP for all control methods, relative to non-high income households.

Minnesota (MN) is reference state for all state specifications. Reference groups for the other variables, are, in order of appearance: age ≤ 50, college education, low income, female, low perceived prevalence, low perceived seriousness, low perceived likelihood of illness, and no past diagnosis.

**Table B.3:** Binary outcome models for support of a publicly funded community-level tick control program.

|  | **Model** | | | | |
| --- | --- | --- | --- | --- | --- |
|  | **A** | **B** | **C** | **D** | **E** |
| Price | -0.028^***^ | -0.029^***^ | -0.03^***^ | -0.03^***^ | -0.03^***^ |
| Age > 50 |  | 0.031 | 0.009 | 0.005 |  |
| Elementary/Middle/HS |  | -0.433^***^ | -0.426^***^ | -0.419^***^ | -0.424^***^ |
| High Income |  | 0.546^***^ | 0.559^***^ | 0.55^***^ | 0.561^***^ |
| Male |  | 0.012 | 0.041 | 0.042 |  |
| Perceived prevalence |  |  | 0.167^**^ | 0.179^***^ | 0.163^**^ |
| Perceived seriousness |  |  | 0.179^*^ | 0.177^*^ | 0.178^*^ |
| Perceived likelihood |  |  | 0.359^**^ | 0.358^**^ | 0.356^*^ |
| Past diagnosis |  |  | 0.159^**^ | 0.163^*^ | 0.162^**^ |
| MI |  |  |  | -0.035 |  |
| WI |  |  |  | -0.112 |  |
| Constant | 1.509^***^ | 1.487^***^ | 1.237^***^ | 1.287^***^ | 1.259^***^ |
| AIC | 7216 | 7126 | 7098 | 7099 | 7094 |
| BIC | 7229 | 7166 | 7165 | 7179 | 7148 |

^*^ *p*<0.1

^**^ *p*<0.05

^***^ *p*<0.01

Estimates are based on 5,973 ‘yes’ or ‘no’ responses to the community level tick control program, with price taking values of $10, $30, or $50 per year. Responses were stacked and binary logit models run. Column E is the preferred specification based on both Akaike’s information criterion (AIC) and the Bayesian information criterion (BIC). Point estimates are presented as parameters rather than odds ratios because willingness to pay is calculated as the negative of the ratio of the sum of non-price parameters over the price parameter. For interpretation, a negative sign means that the variable lowers the probability of a ‘yes’ answer (e.g., price) and a positive sign means the variable increases the probability of a ‘yes’ (e.g., past diagnosis).

**APPENDIX C:**

**Table C.1:** Counts of respondents per household type for estimation samples

| **Household Type** | **Self-Application (Table 1)** | **Commercial Application (Table 2)** | **Community Program (Table 3)** |
| --- | --- | --- | --- |
| **1** | 28 | 8 | 45 |
| **2** | 75 | 25 | 129 |
| **3** | 22 | 5 | 35 |
| **4** | 3 | 0 | 6 |
| **5** | 61 | 23 | 95 |
| **6** | 3 | 1 | 4 |
| **7** | 7 | 4 | 13 |
| **8** | 7 | 2 | 12 |
| **9** | 243 | 50 | 333 |
| **10** | 11 | 2 | 14 |
| Total Responses | 460 | 120 | 686 |

The household types (1 – 10) included in these estimations are not exhaustive. There are other combinations of types, meaning the numbers shown for each type do not add up to the total number of respondents in the estimation sample. The samples are also different, even within the same household type. Respondents used in Tables 1 and 2 are each mutually exclusive sets, while Table 3 includes most but not all respondents (it does not include people who were not interested in the community option).
